# Age‐Adjusted AFP Interpretation in Neonatal Sacrococcygeal Teratoma: Why Adult Reference Ranges May Mislead

**DOI:** 10.1002/ccr3.73174

**Published:** 2026-07-14

**Authors:** Muhammad Abdullah Awan

**Affiliations:** ^1^ Wah Medical College, National University of Medical Sciences Wah Pakistan

**Keywords:** alpha‐fetoprotein, case report, MRI, newborn infant, sacrococcygeal teratoma, tumor marker

## Abstract

In neonatal sacrococcygeal masses, AFP should be interpreted in neonatal context and followed serially after resection. The case by Argüelles Balas et al. would be strengthened by clarifying AFP interpretation and postoperative AFP kinetics alongside MRI, pathology, and clinical follow‐up.

Dear Editor,

I read with great interest the case image report by Argüelles Balas et al. [1], “Neonatal Sacrococcygeal Mass: From Lipoma to Teratoma,” published in *Clinical Case Reports*. The authors present an instructive neonatal case in which a soft, mobile sacrococcygeal mass initially resembling a benign lipoma was ultimately diagnosed as a mature sacrococcygeal teratoma after careful clinical evaluation, MRI, surgical resection with coccygectomy, and histopathological confirmation [1].

One point that deserves clarification is the interpretation of alpha‐fetoprotein (AFP). The authors correctly state that AFP levels are physiologically elevated in neonates; however, the measured AFP value of 126 μg/L is then presented against a reference range of 0.89–8.78 μg/L and described as supporting suspicion of a germ cell tumor [1]. This wording may unintentionally overemphasize the diagnostic weight of a single AFP value in a neonate. In this age group, AFP interpretation is less informative when detached from neonatal context and is more useful when paired with serial postoperative decline.

This clarification would not weaken the diagnostic message of the case. On the contrary, the strongest evidence in the report remains the multimodal assessment: the clinical appearance of the mass, functional anorectal evaluation, MRI demonstration of a fatty lesion with internal solid nodules and an intrapelvic component, complete resection including coccygectomy, and histopathological confirmation of mature teratoma [1]. AFP should therefore be framed as an adjunctive marker rather than a standalone discriminator between lipoma and teratoma in the neonatal period.

For educational value, future similar case reports could specify the infant's exact age at AFP sampling, whether the laboratory reference interval was neonatal or adult‐based, and the actual serial AFP values after resection until normalization. The authors state that AFP decreased to normal values after tumor removal and that the infant remained asymptomatic with normal bowel function at 10 months [1]. Providing the numerical AFP trajectory would make the follow‐up strategy clearer and would help readers understand how tumor marker monitoring was integrated with ultrasound surveillance and conditional MRI.

The case is clinically valuable because it reminds clinicians not to dismiss apparently benign neonatal sacrococcygeal masses without structured assessment. A concise clarification of AFP interpretation would make the report even stronger by preventing overreliance on adult‐style reference ranges while preserving the main lesson: MRI, surgical‐pathological correlation, and longitudinal follow‐up are central in distinguishing neonatal sacrococcygeal lipoma‐like lesions from teratoma.

Argüelles Balas et al. provide a useful warning that neonatal sacrococcygeal masses may conceal teratoma despite benign clinical appearance. The diagnostic and follow‐up message would be strengthened by presenting AFP with neonatal context and serial postoperative values, while emphasizing MRI and histopathology as the decisive elements of the case.

Sincerely,

Muhammad Abdullah Awan

## Author Contributions

**Muhammad Abdullah Awan:** conceptualization, formal analysis, writing – original draft, writing – review and editing.

## Funding

The author has nothing to report.

## Ethics Statement

The author has nothing to report.

## Consent

The author has nothing to report.

## Conflicts of Interest

The author declares no conflicts of interest.

## Data Availability

Data sharing not applicable to this article as no datasets were generated or analysed during the current study.
